# Presence of Vessel Wall Hyperintensity in Unruptured Arteriovenous Malformations on Vessel Wall Magnetic Resonance Imaging: Pilot Study of AVM Vessel Wall “Enhancement”

**DOI:** 10.3389/fnins.2021.697432

**Published:** 2021-07-21

**Authors:** Laura B. Eisenmenger, Jacqueline C. Junn, Daniel Cooke, Steven Hetts, Chengcheng Zhu, Kevin M. Johnson, Jesse M. Manunga, David Saloner, Christopher Hess, Helen Kim

**Affiliations:** ^1^Department of Radiology, University of Wisconsin–Madison, Madison, WI, United States; ^2^Department of Radiology, Mount Sinai Hospital, New York, NY, United States; ^3^Department of Radiology and Biomedical Imaging, University of California, San Francisco, San Francisco, CA, United States; ^4^Department of Radiology, University of Washington, Seattle, WA, United States; ^5^Department of Medical Physics, University of Wisconsin–Madison, Madison, WI, United States; ^6^Division of Vascular and Endovascular Surgery, Minneapolis Heart Institute, Abbott Northwestern Hospital, Minneapolis, MN, United States; ^7^Department of Anesthesia, University of California, San Francisco, San Francisco, CA, United States

**Keywords:** arteriovenous malformation, unruptured AVM, vessel wall enhancement, MRI, vessel wall imaging

## Abstract

**Purpose:** High-resolution vessel wall magnetic resonance imaging (VW-MRI) could provide a way to identify high risk arteriovenous malformation (AVM) features. We present the first pilot study of clinically unruptured AVMs evaluated by high-resolution VW-MRI.

**Methods:** A retrospective review of clinically unruptured AVMs with VW-MRI between January 1, 2016 and December 31, 2018 was performed documenting the presence or absence of vessel wall “hyperintensity,” or enhancement, within the nidus as well as perivascular enhancement and evidence of old hemorrhage (EOOH). The extent of nidal vessel wall “hyperintensity” was approximated into five groups: 0, 1–25, 26–50, 51–75, and 76–100%.

**Results:** Of the nine cases, eight demonstrated at least some degree of vessel wall nidus “hyperintensity.” Of those eight cases, four demonstrated greater than 50% of the nidus with hyperintensity at the vessel wall, and three cases had perivascular enhancement adjacent to nidal vessels. Although none of the subjects had prior clinical hemorrhage/AVM rupture, of the six patients with available susceptibility weighted imaging to assess for remote hemorrhage, only two had subtle siderosis to suggest prior sub-clinical bleeds.

**Conclusion:** Vessel wall “enhancement” occurs in AVMs with no prior clinical rupture. Additional studies are needed to further investigate the implication of these findings.

## Introduction

Traditional vascular imaging has been primarily “lumenography,” or imaging techniques that delineate the vascular lumen to study vascular pathology. These conventional techniques include digital subtraction angiography (DSA), computed tomography angiography (CTA), and magnetic resonance angiography (MRA) (Mandell and Shroff, 2011; Dieleman et al., 2014; Matouk et al., 2016), focusing attention to the inside of blood vessels; however, it has long been appreciated that cerebrovascular disease pathogenesis resides, in large part, within the vessel wall. High-resolution vessel wall magnetic resonance imaging (VW-MRI) represents an innovative method to evaluate the intracranial vessel wall in both healthy vessels and vascular disease.

Initially, the majority of studies were focused on steno-occlusive cerebrovascular disease such as intracranial atherosclerosis (Vergouwen et al., 2011; Skarpathiotakis et al., 2013; Mossa-Basha et al., 2015), primary central nervous system vasculitis (Mandell et al., 2012; Obusez et al., 2014; Mossa-Basha et al., 2015), reversible cerebral vasoconstriction syndrome (Mandell et al., 2012; Obusez et al., 2014; Mossa-Basha et al., 2015), drug-induced vasculopathies (Han et al., 2008), and intracranial dissections (Chung et al., 2014; Natori et al., 2014). More recently, there has been increased enthusiasm for the utilization of VW-MRI to further characterize vascular malformations, with intracranial aneurysms being the most commonly studied lesion (Matouk et al., 2013, 2016; Edjlali et al., 2014; Nagahata et al., 2016; Tian et al., 2019; Wang et al., 2019; Liu et al., 2020; Zhu et al., 2020). Current evidence suggests that vessel wall hyperintensity on post-contrast VW-MRI, often referred to as vessel wall “enhancement,” is associated with ruptured (Matouk et al., 2013; Nagahata et al., 2016; Wang et al., 2019), symptomatic (Zhu et al., 2020), and unstable (Edjlali et al., 2014; Edjlali et al., 2018) aneurysms. Given the early evidence that VW-MRI may help in the identification of high risk aneurysms, VW-MRI could also be a tool to improve the identification and characterization of high risk intracranial arteriovenous malformations (AVMs); however, fewer VW-MRI studies have been performed on these more complex vascular malformations, now limited to two case series (Matouk et al., 2016; Petridis et al., 2018) and a few case reports (Omodaka et al., 2015; Komatsu et al., 2018; Bhogal et al., 2019) in only ruptured AVMs. While these reports did document vessel wall enhancement within the ruptured AVMs, it is unknown if unruptured AVMs also enhance as no studies to date have evaluated unruptured AVMs with this technique (Omodaka et al., 2015; Matouk et al., 2016; Komatsu et al., 2018; Bhogal et al., 2019). We present the first pilot study of VW-MRI in clinically unruptured brain AVMs and discuss this method’s promise and limitations when applied to this type of complex vascular malformation.

## Materials and Methods

A search for AVMs at UCSF with VW-MRI between January 1, 2016 and December 31, 2018 was performed with nine cases of clinically unruptured AVMs identified. IRB approval was obtained to review the images retrospectively.

### Imaging Protocol

Images for the following cases were acquired at a 3T whole-body MR scanner (GE MR750) with a standard eight-channel head coil. A T1-weighted 3D fast-spin-echo sequence (CUBE) obtained in the sagittal plane with variable refocusing flip angle was acquired both pre- and post-contrast. The parameters were: non-selective 90° excitation; TR/TE = 1000/17 ms; field of view = 18 cm × 18 cm; matrix = 300 × 300; slice thickness = 0.6 mm; number of slices 288; voxel size = 0.6 mm isotropic; echo train length of 60; ARC acceleration factor of two in phase encoding direction; scan time = 7 min 55 s, within the imaging acquisition recommendations defined by the Vessel Wall Imaging Study Group of the American Society of Neuroradiology for intracranial vessel wall imaging (Mandell et al., 2017). Post-contrast CUBE was acquired approximately 5 min after the injection of Magnevist^®^ (gadopentetate dimeglumine) with a dose of 0.2 mL per kg of body weight.

### Imaging Review

A consensus review was obtained by two board certified neuroradiologists with expertise in vascular, and specifically vessel wall, imaging (8 and 19 years of experience). The reviewers were blinded to patient history and demographics. If available, T2^∗^ gradient echo images were reviewed for the evidence of old hemorrhage (EOOH) indicated by areas of low signal adjacent to the AVM. Post-contrast VW-MRI images were reviewed for the presence or absence of vessel wall “hyperintensity” on post-contrast images within the nidus, defined as greater hyperintensity than adjacent normal arterial vessel segments. Pre-contrast VW-MRI images were reviewed for intrinsic T1 hyperintensity to confirm that hyperintensity on post-contrast imaging was due to contrast administration. Axial, coronal, and sagittal VW-MRI images were reviewed to estimate the percent of the nidal vessels with post-contrast nidal vessel wall hyperintensity. The percent volume of nidal vessel wall “hyperintensity” was approximated into five groups: 0% or absence, 1–25, 26–50, 51–75, and 76–100%. The presence or absence of perivascular enhancement was also assessed in the regions surrounding the nidus, defined as post-contrast enhancement outside of the vasculature but within 2 mm of the external aspect of the vessel wall. The original radiologic clinical reports were reviewed for content including if the reading radiologist mentioned the presence or absence of vessel wall and perivascular “enhancement.” The Spetzler-Martin grade was recorded.

## Results

**FIGURE 1:**
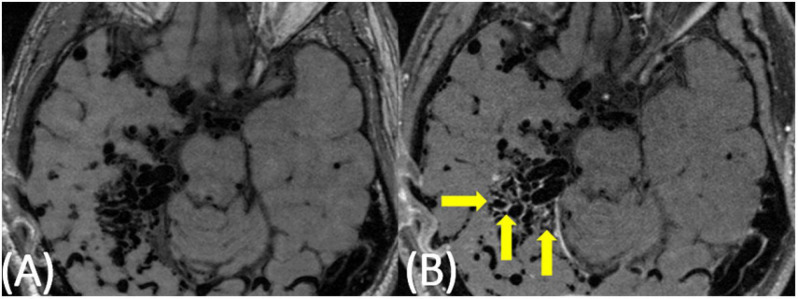
51-year-old man with a large AVM centered in the right temporoparietal lobe. On pre-contrast VW-MRI **(A)**, no intrinsic T1 hyperintensity is present. On post-contrast VW-MRI **(B)**, there were multiple areas of hyperintensity at the vessel wall within the nidus (yellow arrows).

**FIGURE 2:**
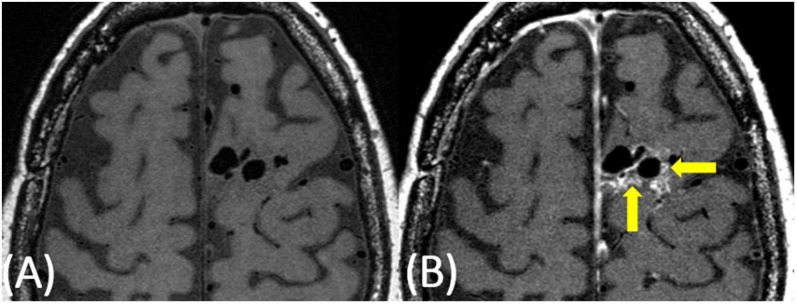
63-year-old female with a predominantly left frontal AVM. On pre-contrast VW-MRI **(A)**, no intrinsic T1 hyperintensity is present. On post-contrast VW-MRI **(B)**, there were multiple areas of hyperintensity at the vessel wall as well as perivascular hyperintensity within the nidus (yellow arrows).

**FIGURE 3:**
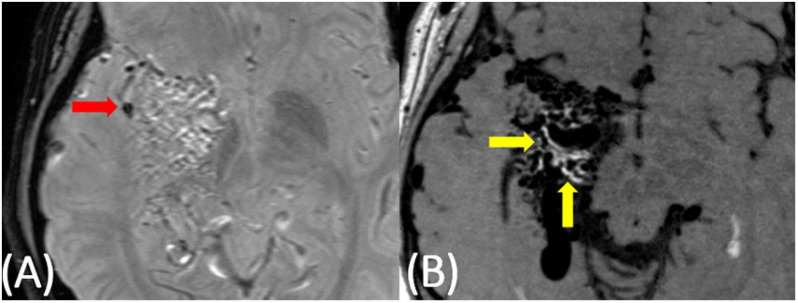
20-year-old female with a right temporoparietal AVM with a small area of sub-clinical evidence of prior hemorrhage on susceptibility-weighted imaging (**A**, red arrow). On post-contrast VW-MRI **(B)**, there were multiple areas of hyperintensity at the vessel wall (yellow arrows).

Nine subjects with clinically unruptured AVMs were identified. Subject demographics and AVM details are presented in Table 1. No subjects had a history of vasculitis or other systemic inflammatory process. Five subjects were female and four were male ranging in age from 24 to 63 with an average age of 39. Of the nine cases, eight demonstrated at least some degree of hyperintensity on post-contrast VW-MRI at the vessel wall within the nidus (Figure 1 and Supplementary Figures 1, 2). Of those eight cases, four demonstrated greater than 50% of the nidus with hyperintensity at the vessel wall, and three cases had perivascular enhancement adjacent to nidal vessels (Figure 2). The original radiologic reports agreed with the consensus reviewers’ assessment of the presence or absence of vessel wall and perivascular “enhancement” in all cases. Although none of our cases had prior clinically apparent hemorrhage/AVM rupture, of the six patients with available susceptibility weighted imaging to assess for subtle, remote hemorrhage, only two had a small degree of siderosis to suggest prior sub-clinical bleeds (Figure 3).

**TABLE 1:** Arteriovenous malformation (AVM) case demographics and characteristics.

	**Sex**	**Age**	**Presenting symptoms**	Location*	**S-M Grade^∝^**	**EOOH^◆^**	**% Nidus hyperintensity**	**Perivascular hyperintensity**
Case 1	Male	51	Seizures, migraines, pain over right eye	Right posterior temporal	IV	Absent	76–100%	Present
Case 2	Female	63	Seizure	Left frontal	III	Absent	76–100%	Present
Case 3	Female	30	Headaches, blurred vision, occipital fullness	Left precuneus	I	Present	1–25%	Absent
Case 4	Female	20	Left homonymous hemianopsia	Right mesial temporal	V	Present	51–75%	Present
Case 5	Male	24	Incidentally found	Left frontoparietal	III	Absent	51–75%	Absent
Case 6	Male	49	Incidentally found	Left medial parietal	II	NA	1–25%	Absent
Case 7	Male	62	Seizure	Left central sulcus	IV	Absent	26–50%	Absent
Case 8	Female	53	Headaches, visual symptoms, imbalance	Left occipital lobe	IV	NA	1–25%	Absent
Case 9	Female	43	Headaches	Right pre- and post-central gyrus	III	NA	0%	Absent

*Location where the AVM is primarily centered.

*∝Spetzler-Martin (S-M) grade of the AVM.*

*◆Evidence of Old Hemorrhage (EOOH): foci of siderosis suggesting prior hemorrhage, in these cases all sub-clinical.*

## Discussion

To our knowledge, our pilot study is the first to specifically evaluate for and demonstrate the presence and extent of vessel wall hyperintensity, often referred to as vessel wall “enhancement,” on black blood, high-resolution VW-MRI in cases of clinically unruptured AVMs. We found eight of nine cases demonstrated nidal “enhancement,” and three of those eight demonstrated perivascular “enhancement” adjacent to the nidus. Other than our study, there are limited publications evaluating AVMs using VW-MRI. The largest by Matouk et al. (2016) reported their findings on 13 ruptured brain AVMs concluding that VW-MRI was useful in identifying the site-of-rupture in patients with ruptured brain AVMs, although it could not do so in all patients. The authors also noted that VW-MRI was useful in demonstrating the precise spatial relationship of blood products adjacent to angioarchitectural vascular structures, thereby helping to further target attention to areas of potential interest. Thick vessel wall enhancement was demonstrated in all ruptured vascular structures in their series; however, the authors did caution that multiple components of the AVMs demonstrated mesh-like and complex, flow-related enhancement (Matouk et al., 2016). One case series did evaluate black blood MRI in ruptured and unruptured AVMs finding “enhancement” in the nidus of five out of six unruptured AVMs (Petridis et al., 2018). The authors suggest that this may be due to inflammation; however, this study did not evaluate the extent of nidal involvement, and the black blood sequence used had a resolution of 0.9 mm (Petridis et al., 2018), lower than what is recommended for VW-MRI (Mandell et al., 2017). Even though several review articles on VW-MRI have also anecdotally commented on the potential use of VW-MRI in intracranial AVMs (Young et al., 2019), we found only three additional case reports with the use of VW-MRI, all of which had rupture sites involving intranidal aneurysms that demonstrated associated vessel wall “enhancement” (Omodaka et al., 2015; Komatsu et al., 2018; Bhogal et al., 2019). Prior to our pilot study, no study has evaluated the presence and extent of vessel wall hyperintensity, or “enhancement,” in this high a number of clinically unruptured AVMs. In addition, our study is the first in unruptured AVMs to follow the American Society of Neuroradiology’s vessel wall imaging recommendations regarding the VW-MRI sequence parameters used for evaluating the intracranial vessels (Mandell et al., 2017).

Vessel wall magnetic resonance imaging has been more extensively used to study intracranial aneurysms, identifying the site of aneurysm rupture (Matouk et al., 2013; Kondo et al., 2014; Hu et al., 2016; Nagahata et al., 2016) as well as unstable, symptomatic aneurysms (Edjlali et al., 2014; Edjlali et al., 2018; Zhu et al., 2020) and sites of aneurysm inflammation (Hu et al., 2016; Larsen et al., 2018). Although intracranial AVMs are much more complex shunting vascular malformations, one may speculate that areas of enhancement within AVMs may also be indicative of high risk features. This is relevant to patient care and risk stratification as the decision to treat unruptured brain AVMs remains controversial, especially after the publication of the ARUBA trial (A Randomized Trial of Unruptured Brain AVMs) and SIVMS (Scottish Intracranial Vascular Malformation Study) prospective, population-based cohort study (Al-Shahi Salman et al., 2014; Mohr et al., 2014). Both studies concluded that a conservative, non-interventional approach was associated with the best clinical outcomes; however, the methods and results of both studies remain highly controversial with many experts feeling these studies unfairly portrayed treatment outcomes as compared to watchful waiting (Elhammady and Heros, 2017; Magro et al., 2017). Philosophically, the guiding principle for any treatment strategy is the achievement of the desired result—in this case AVM obliteration—while posing minimal harm to the patient in order to provide improved outcomes. Methods of AVM treatment include surgical resection, Gamma Knife radiosurgery, embolization, and combinations of these methods, often in a staged manner overtime; however, these treatments are not without significant morbidity and, in some cases, mortality (Matouk et al., 2013). It stands to reason that identification of higher risk AVMs, as well as specific high risk AVM features, would prove to be instrumental in the improved risk stratification of these lesions for treatment and better patient outcomes. VW-MRI could be another tool in the characterization of AVMs.

Although many studies support the relationship between vessel wall “enhancement” and vessel rupture and/or instability, several complicating factors exist in the imaging of vascular lesions. For one, some studies are now finding that a proportion of vascular malformations that enhance demonstrate persistent and stable “enhancement” over time with no symptomatology or subsequent rupture (Tian et al., 2019). This persistent hyperintensity on imaging could indicate remodeled vessel wall without active inflammation as well as true persistent inflammatory changes; however, more studies and larger, longitudinal studies are needed to investigate if symptomology, rupture, and/or mortality are associated with vessel wall hyperintensity as well as histopathologic correlation with sites of enhancement versus non-enhancement. Future longitudinal VW-MRI studies evaluating unruptured AVMs should follow untreated patients over several years to truly understand the implications of AVM “enhancement” presence and extent on AVM risk and patient outcomes. Apparent vessel wall “enhancement” can also be misinterpreted from vasa vasorum, adjacent veins, and endovascular interventions such as mechanical thrombectomy or embolization causing altered flow (Mandell et al., 2017); however, maybe one of the hardest things to discern is the contributions of the VW-MRI sequences themselves to the appearance of vessel wall hyperintensity.

The third main complicating factor that can occur during the imaging of vascular lesions relates to complex blood flow within the area of abnormality. Vessel wall imaging requires high contrast-to-noise ratio (CNR) and spatial resolution in addition to blood flow and cerebral spinal fluid (CSF) suppression, yielding the appearance of black blood which allows for the clear visualization of the vessel wall. Some of the most commonly employed VW-MRI sequences are variable refocusing flip angle (VRFA) sequences with T1 or proton density-weighted pre- and post-contrast imaging (VISTA; Philips Healthcare), sampling perfection with application-optimized contrasts by using different flip angle evolutions (SPACE; Siemens), and CUBE software (GE Healthcare) (Young et al., 2019). While VRFA sequences are adequate in most cases, because gadolinium shortens T1 relaxation time, “black blood” suppression in areas of abnormal or even normal in-plane flow can be diminished, potentially causing unsuppressed contrast in flowing blood, artifactually appearing as vessel wall contrast “enhancement.” Examples of this are seen with turbulence and recirculation within aneurysms, slow flow within dilated lumens, and retrograde filling of distal collateral branches with proximal occlusion (Mandell et al., 2017; Tian et al., 2019); however, this altered blood flow leading to potential incomplete suppression on VFRA sequences may in and off itself be a high risk feature, such as areas of low wall sheer stress in aneurysms (Boussel et al., 2008), although this hypothesis has yet to be well investigated. Preparation pulses can be helpful in cases where this flow-related artifact is occurring, such as motion-sensitized driven equilibrium (MSDE) which uses flow-sensitive dephasing gradients (Young et al., 2019). Another approach to optimize blood and CSF suppression is delayed alternating nutation for tailored excitation (DANTE), a preparation pulse that uses a series of low flip angle non-selective pulses interleaved with gradient pulses of short repetition times (Mossa-Basha et al., 2016; Fan et al., 2017; Mandell et al., 2017; Tan et al., 2018). In the current pilot study, the imaging was obtained in the clinical setting and evaluated retrospectively; therefore, while the VW-MRI sequence parameters follow the American Society of Neuroradiology’s intracranial vessel wall imaging recommendations, no additional blood suppression sequences were compared to the CUBE imaging. Ideally, future studies would confirm our findings as well as compare “enhancement” presence and extent utilizing different VW-MRI sequences in AVMs.

These complicating factors are likely to be even more confounding in cases of intracranial AVMs given the higher rates of blood flow in the feeding arteries, the more complex network of abnormal vessels in the AVM nidus, the potential presence of intranidal aneurysms and/or pseudoaneurysms, as well as altered morphology and blood flow in the draining veins. While the previous case series and case reports focus on sites of AVM rupture (Omodaka et al., 2015; Matouk et al., 2016; Komatsu et al., 2018; Petridis et al., 2018; Bhogal et al., 2019), our brief report highlights the complexity of vessel wall hyperintensity patterns in AVMs, and not in ruptured AVMs, but in clinically unruptured lesions. If AVM data are found to be similar to aneurysm VW-MRI data, that may mean AVMs with vessel wall “enhancement” are more unstable and require a new paradigm in AVM risk classification. In addition, although all cases in our study were clinically unruptured, two of six with susceptibility-weighted imaging demonstrated evidence of subclinical old hemorrhages. Our findings illustrate the need for more extensive research in these cases as well as the need for longitudinal monitoring of VW-MRI signal within brain AVMs, evaluation for both clinically evident and subclinical hemorrhage through susceptibility-weighted imaging, and the correlation of imaging findings with histopathologic analysis.

Our study does have multiple limitations. First, due to the retrospective nature of this case series, the VW-MRI sequence utilized was the variable refocusing flip angle CUBE (GE Healthcare) sequence, which does not have any additional suppression pulses to assist in the elimination of flow-related artifacts or other causes of incomplete blood signal suppression. Second, these are cases of intracranial AVMs with no prior clinical rupture. While a number of our cases had susceptibility-weighted MRI sequences acquired to confirm the absence of only prior hemorrhage (lack of hemosiderin staining), a couple of the cases do not. Future prospective studies should include susceptibility-weighted imaging in all cases. Third, given the small number of subjects and lack of long-term follow-up, it is hard to derive any meaningful conclusions regarding the implications of the AVM “enhancement.” Larger, longitudinal studies are needed to discern if there is any increased risk of symptomology, rupture, and/or mortality associated with the presence, amount, degree, or pattern of vessel wall hyperintensity. In addition, histopathologic correlation with sites of enhancement versus non-enhancement is needed to fully understand the findings of both our pilot study of clinically unruptured AVMs and the prior reports of ruptured cases; however, our case series is essential in that it highlights the complexity of these lesions and the need for this type of AVM research.

## Conclusion

To our knowledge, this is the largest pilot study of clinically unruptured AVMs evaluated by high-resolution VW-MRI, demonstrating varying degrees of vessel wall hyperintensity, or “enhancement,” in the majority of the cases. While a portion of the hyperintensity could be due to incomplete blood suppression, given the high percentage of involvement within the nidus in several of the cases, this brief report illustrates that vessel wall enhancement occurs even in AVMs with no prior rupture. Larger, longitudinal studies are needed to more fully understand the implications of this intranidal “enhancement” including whether or not the presence, degree, morphology, and/or location of the hyperintensity has any increased risk of morbidity or mortality. Our findings illustrate the need for more extensive research in these cases including future studies with histopathologic analysis.

## Data Availability Statement

The original contributions presented in the study are included in the article/Supplementary Material. Further inquiries can be directed to the corresponding author/s.

## Ethics Statement

The studies involving human participants were reviewed and approved by the UCSF IRB. Written informed consent for participation was not required for this study in accordance with the national legislation and the institutional requirements.

## Author Contributions

LE, JJ, DC, SH, CZ, KJ, JM, DS, CH, and HK contributed to the study design. LE and JJ collected the data. LE wrote the first draft. All authors contributed to editing and drafting of the final draft and agreed with the submitted version.

## Disclaimer

The content is solely the responsibility of the authors and does not necessarily represent the official views of the NIH.

## Conflict of Interest

The authors declare that the research was conducted in the absence of any commercial or financial relationships that could be construed as a potential conflict of interest.

## Supplementary Material

Supplementary Figure 1 30-year-old female with a predominantly left occipital AVM. On pre-contrast VW-MRI **(A)**, no intrinsic T1 hyperintensity is present. On post-contrast VW-MRI **(B)**, there was only one area of hyperintensity near the vessel wall (yellow arrow). No perivascular hyperintensity was present.

Supplementary Figure 2 24-year-old male with a left frontoparietal AVM. On pre-contrast VW-MRI **(A)**, no intrinsic T1 hyperintensity is present; however, there were multiple areas of hyperintensity at the vessel wall (yellow arrows) on post-contrast VW-MRI **(B)**. No perivascular hyperintensity was present.
