# Supplementary figures and images for: Presence of Vessel Wall Hyperintensity in Unruptured Arteriovenous Malformations on Vessel Wall Magnetic Resonance Imaging: Pilot Study of AVM Vessel Wall “Enhancement”
Source: Front Neurosci. 2021 Jul 21;15:697432. doi: 10.3389/fnins.2021.697432 (PMC8334001; doi:10.3389/fnins.2021.697432)

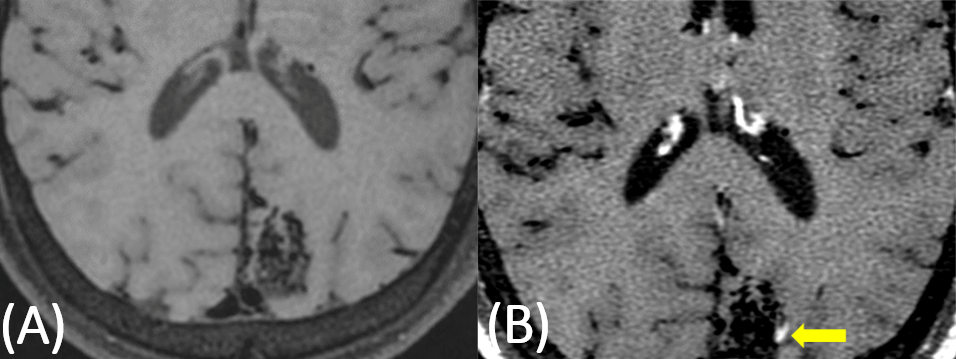

Supplement: Supplementary Figure 1 — 30-year-old female with a predominantly left occipital AVM. On pre-contrast VW-MRI (A), no intrinsic T1 hyperintensity is present. On post-contrast VW-MRI (B), there was only one area of hyperintensity near the vessel wall (yellow arrow). No perivascular hyperintensity was present. [file Image_1.TIF]

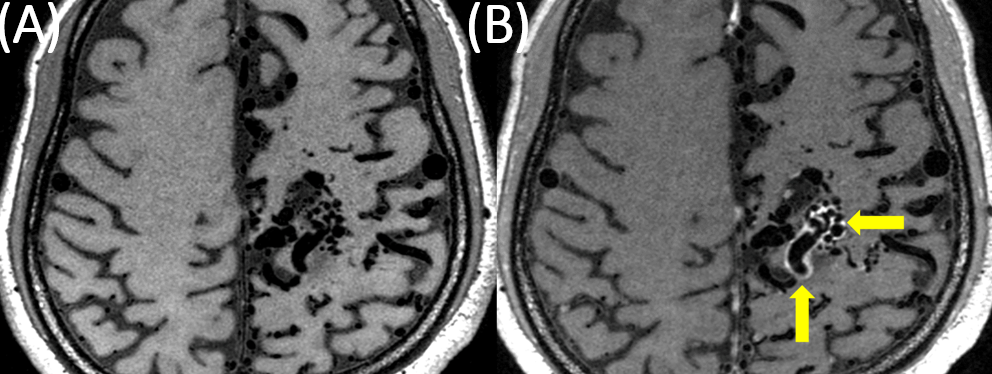

Supplement: Supplementary Figure 2 — 24-year-old male with a left frontoparietal AVM. On pre-contrast VW-MRI (A), no intrinsic T1 hyperintensity is present; however, there were multiple areas of hyperintensity at the vessel wall (yellow arrows) on post-contrast VW-MRI (B). No perivascular hyperintensity was present. [file Image_2.TIF]
